# The origin of rare alkali metals in geothermal fluids of southern Tibet, China: A silicon isotope perspective

**DOI:** 10.1038/s41598-019-44249-5

**Published:** 2019-05-27

**Authors:** Wei Wang, Hai-Zhen Wei, Shao-Yong Jiang, Hong-Bing Tan, Christopher J. Eastoe, Anthony E. Williams-Jones, Simon V. Hohl, He-Pin Wu

**Affiliations:** 1 State Key Laboratory of Geological Processes and Mineral Resources, School of Earth Resources, China University of Geosciences, Wuhan, 430074 P.R. China; 2 State Key Laboratory for Mineral Deposits Research, School of Earth Sciences and Engineering, Nanjing University, Nanjing, 210023 P.R. China; 3 School of Earth Sciences and Engineering, Hohai University, Nanjing, 210098 P.R. China; 4 Department of Geosciences, University of Arizona, Tucson, Arizona 85721 United States; 5 Department of Earth and Planetary Sciences, McGill University, Montreal, H3A 0E8 Canada

**Keywords:** Hydrogeology, Geochemistry

## Abstract

Geothermal waters from the Semi, Dagejia and Kawu hot springs in the Shiquanhe-Yarlung Zangbo geothermal field of southern Tibet (China) are highly enriched in rare alkali metals (RAM). However, the enrichment mechanism is still hotly debated. Here, we report the first silicon isotope data of these geothermal waters to unravel the origin of the extreme RAM enrichments. Sinter precipitation in the spring vents and water-rock interaction in the deep reservoir controlled both the silicon budget and silicon isotope fractionation. The rates of water-rock interaction and sinter precipitation in three spring sites decrease in the sequences Semi > Kawu > Dagejia, and Dagejia > Kawu > Semi respectively. Silicon isotope fractionation during sinter precipitation (i.e. Δ^30^Si_precipitate-solution_ < −0.1‰) is less than that due to water-rock interaction (i.e. Δ^30^Si_solution-rocks_ at least as high as −0.47‰), which makes it possible to use the δ^30^Si signatures of springs to evaluate the intensity of water-rock interaction. Based on the available evidence, a conceptual model of RAM enrichment is proposed: (i) persistent magmatic activity in southern Tibet provided the initial enrichment of the RAM in host rocks and a heat sources for the deep reservoirs of geothermal systems; (ii) the high Cl^−^ content and long residence time (thousands of years) promote the leaching of RAM from the silicate host rocks.

## Introduction

The Mediterranean-Himalayan geothermal belt in southern Tibet is one of the most active geothermal regions in the world. In this region, precipitates from hydrothermal springs display extreme enrichments in rare alkali metals (RAM) and boron, exemplified by rare geyserite Cs-deposits at the Dagejia, Semi and Gulu sites^1^. Geothermal waters at these sites are also characterized by abnormally high boron content (up to 450 ppm) and low δ^11^B values (−16.6 to −10.9‰)^2^, which is in stark contrast with the Yellowstone (USA) geothermal system, where the geothermal water contains 0.46 to 29.08 mg/L of boron with δ^11^B of −9.3 to +4.4‰^3^. The data for the Tibetan sites reflect the contribution of residual magma degassing, which is confirmed by the He isotope signature^4^. Owing to their highly incompatible nature, the RAM and boron are concentrated in the magma during partial melting and/or fractional crystallization, and consequently their abundances typically increase from primitive ultramafic rocks to highly evolved felsic rocks, and ultimately to magmatic volatiles. Significantly, the RAM in the magmatic volatiles phase are enriched by 10^1^ to 10^3^ times relative to the bulk melt composition^5^. Previous studies have proposed that collisional orogeny promoted partial melting of the upper crust of the Qinghai-Tibet Plateau and that fractional crystallization of the magma and the segregation of magmatic volatiles led to formation of RAM-enriched pegmatite^1^, and other RAM-enriched intrusive phases leached by hydrothermal fluid^6^. However, whether the RAM enrichment in the geothermal springs is from residual magma degassing or from leaching of RAM-enriched host rocks is still hotly debated due to the lack of convincing evidence for either hypothesis.

Recent studies have suggested that silicon isotopes may serve as a sensitive tracer for bio-physicochemical and ore-forming processes in terrestrial and oceanic environments and the Earth’s interior^7–9^. There have been very few studies of silicon isotopes behavior, however, in geothermal systems^10–14^, and these have focused mainly on the diagenetic and biogenic effects on silicon fluxes and isotopic compositions. Here, we present a systematic silicon isotope study of three geothermal spring sites near the Yarlung Zangbo Suture Zone, with the main aim of constraining the factors responsible for the exceptional enrichment of RAM in the geothermal water, through developing an understanding of the relationship between silicon isotope fractionation, reservoir temperature and water chemistry. The subsidiary aims are to determine whether siliceous sinter formation (and/or water-rock interaction) control silicon isotope fractionation in such systems and to investigate the potential for using δ^30^Si as an indicator of intensity of geothermal water-rock interaction. We conclude with a conceptual model for the enrichment of the RAM and boron in the geothermal waters.

**Figure 1:**
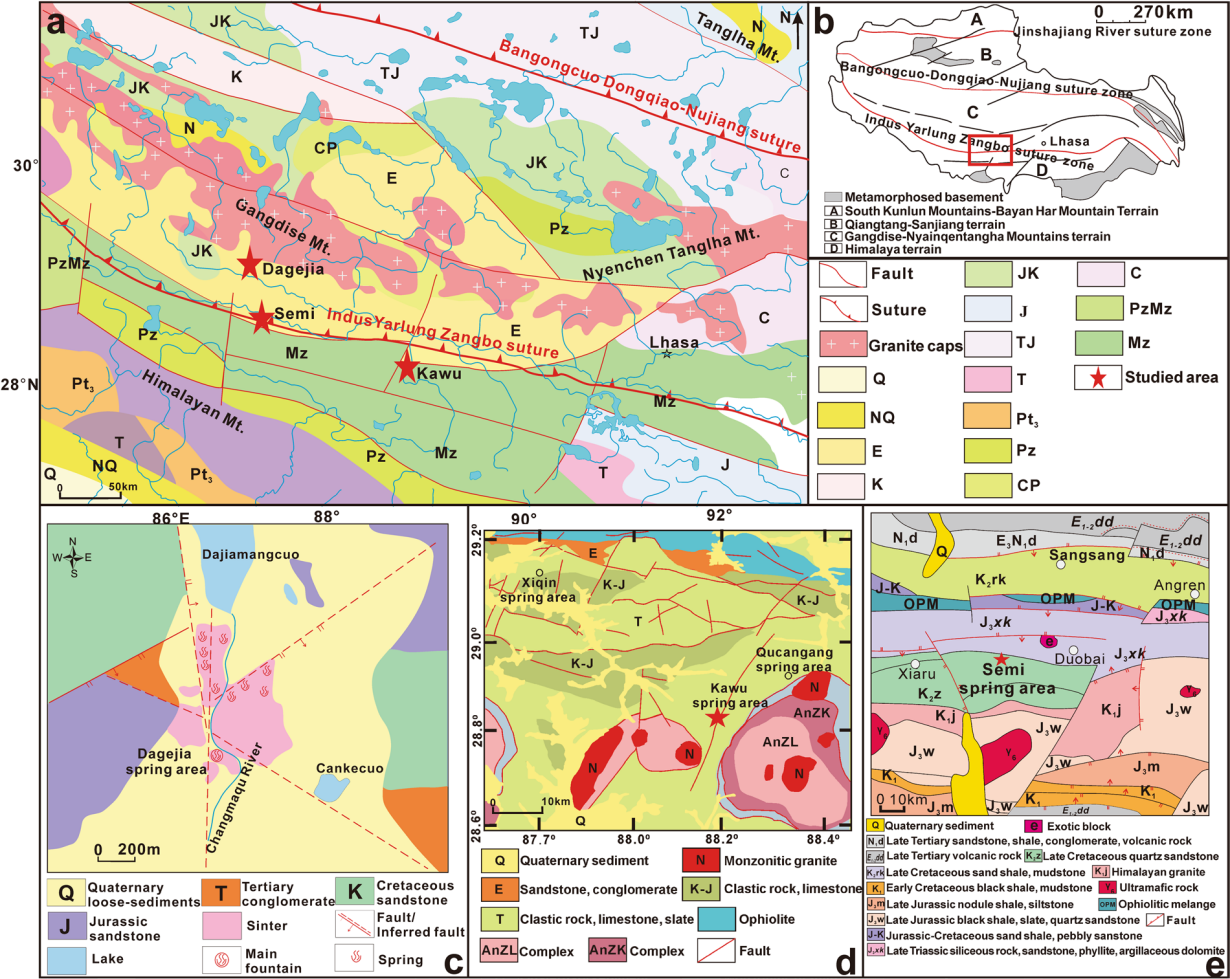
(**a**) A geological map of southern Tibet, China, showing the locations of the Dagejia, Semi, Kawu spring sites (modified after Tan *et al*.^45^). (**b**) The location of major tectonic structures in the region. (**c**–**e**) Geological maps of the areas surrounding the Dagejia, Kawu and Semi geothermal sites (modified after Zheng *et al*.^1^).

Geologically, the Tibetan plateau is located at the junction between the Eurasian and Indian plates, and consists of four east-west-trending continental terranes, from north to south: the South Kunlun Mountains-Bayan Har Mountain Terrane, the Qiangtang-Sanjiang Terrane, the Gandise-Nyainqentangha Mountains Terrane (Lhasa Terrane) and the Himalaya Terrane (Fig. 1a,b). The terranes are separated by three suture zones: the Jinshajiang River Suture Zone, the Bangongcuo-Dongqiao-Nuojiang Suture Zone and the Indus Yarlung Zangbo Suture Zone. The most intense geothermal activity occurs in the Lhasa Terrane near the Indus Yarlung Zangbo Suture Zone, on the southern margin of the Gangdise Magmatic Arc^6^. This part of the Terrane is dominated by the Cretaceous-Tertiary Gangdise Batholith and the Paleogene Linzizong volcanic succession which are accompanied by minor Triassic-Cretaceous volcano-sedimentary rocks^15^. The geothermal spring sites considered in this work are on the southern side of Gangdise Magmatic Arc, which is of Cretaceous-Tertiary age^2^. The temperatures of hot springs and the reservoir distributed along the Indus Yarlung Zangbo Suture Zone range from 35 to 89 °C and from 47 to 227 °C^2^, respectively.

The Dagejia site is located closest to the Gangdise arc, in an area underlain by unconsolidated Quaternary sediments (loess) and siliciclastic sedimentary rocks (conglomerate and sandstone) ranging from Tertiary to Jurassic in age, and Cretaceous magmatic rocks (mainly biotite monzonitic granite, hornblende monzonitic granite) (Fig. 1c). Modern siliceous sinter is widespread. The Kawu site is located to the south of Yarlung Zangbo Suture, in an area dominated by unconsolidated Jurassic-Tertiary sedimentary rocks. Outcrops are sparse in the vicinity of the site, expose a single lithotype, namely Cretaceous gneissic monzonitic granite (Fig. 1d)^16^. The Semi site is located intermediately to the north of the Indus Yarlung Zangbo Suture Zone and, in contrast to the other geothermal systems, the area is underlain by a diverse assemblage of rock-types that includes ultramafic rocks, tourmaline granite, and late Triassic slate, quartzite and phyllite. The siliceous sinter is better developed than around the Kawu site, although it is thinner (≤5 m in thickness) and less extensive than that the Dagejia site (Fig. 1e)^1^.

## Results

**Figure 2:**
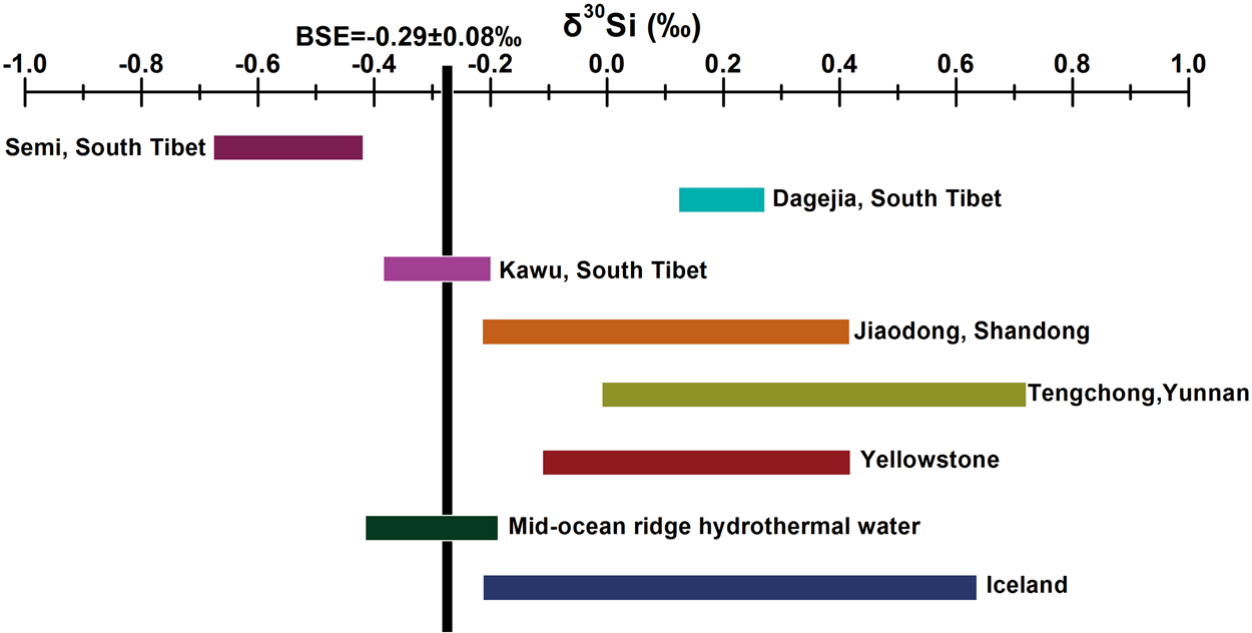
Silicon isotope values in geothermal springs worldwide^10–13,29,30,32^ and from this study (southern Tibet).

The temperatures of the geothermal waters at the three sites were between 70 and 90 °C and the temperature of nearby stream water was 15 °C. Most of the waters have near neutral pH (7.0 to 8.7); the highest pH was measured at the Semi site. The geothermal waters were classified into two types based on their chemical compositions (Table S1), namely a Na-HCO_3_^−^ type with lower concentrations of SiO_2_, B, and RAM (at Dagejia) and a Na-Cl^−^ type with higher concentration of SiO_2_, B, and RAM (at Kawu and Semi). The highest concentrations of SiO_2_ (200 ppm), Cl (900 ppm), B (400 ppm), Li (30 ppm) and Cs (50 ppm) were measured in samples from the Semi spring; these are up to several to hundreds of times greater than in the adjacent stream waters. Modern siliceous sinter was only observed and sampled at the Dagejia site. Their chemical compositions of spring-vent sinter at Dagejia site are given in Table S2. According to the XRD spectra, the siliceous sinter is of two types, Opal-A and Opal-CT. The contents of rare alkali metals (e.g., Cs) varies from 2000 ppm to 10000 ppm, which is hundreds of times higher than that in the average upper crust. The δ^30^Si values of the geothermal water ranges from −0.67‰ to +0.25‰ (−0.37‰ to −0.21‰ at Kawu, +0.13‰ to +0.25‰ at Dagejia and −0.67‰ to −0.44‰ at Semi) and are generally more negative than those of geothermal water elsewhere (see Table S3, Fig. 2). The stream water displays a wider range of δ^30^Si values, i.e., from −0.79‰ to +0.54‰; the lowest value (−0.79‰) is for a sample from the Dagejia stream and the highest (+0.54‰) is for the Yarlung Zangbo river. The silicon isotope values of modern sinter at the Dagejia site are relatively enriched in the light ^28^Si isotope (from −1.80‰ to +0.09‰). The average silicon isotope value of Opal-A is −0.24 ± 0.24‰ (n = 2), and that of Opal-CT is −1.75 ± 0.10‰ (n = 2) (Table S3).

### Water-rock interaction in the deep reservoir

**Figure 3:**
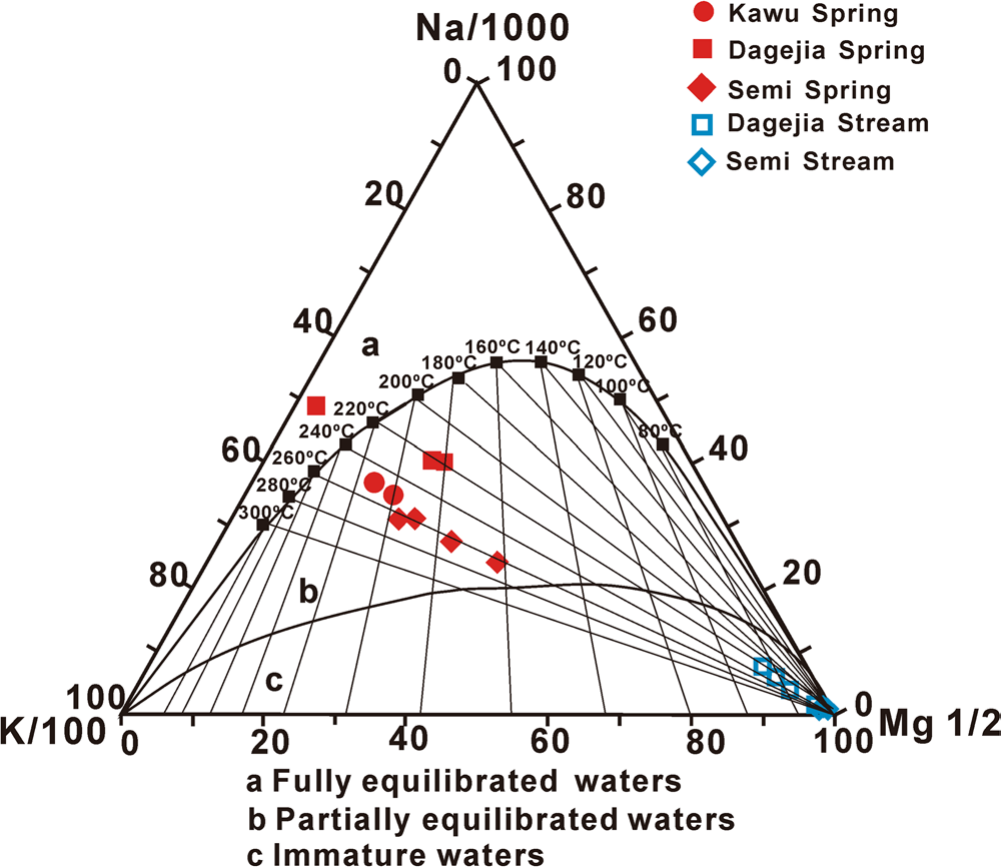
Distribution of aqueous samples on the Na/1000-K/100-Mg^1/2^ ternary diagram.

The temperature of a subsurface thermal water reservoir is a key parameter in evaluating geothermal resources^17^. A variety of chemical geothermometers have been proposed, including the quartz thermometer^18^, the chalcedony thermometer^19,20^, the Na-K thermometer^21,22^ and the Na-K-Ca thermometer^23^. Because of the rapid convection of geothermal water to the surface, the chemical geothermometers report the highest temperatures in the geothermal reservoirs. Reservoir temperatures estimated from these geothermometers are presented in Table S4. Sharp decreases in temperature and pressure near the discharge points result in over-saturation of the aqueous solution and the precipitation of silica. Consequently, the temperatures estimated by the quartz and chalcedony geothermometers, from 50 to 160 °C, indicate the discharge temperatures rather than the reservoir temperatures. For the same reason, the precipitation of calc-sinter (i.e. tufa) near the surface would adversely affect application of the Na-K-Ca thermometer in determining reservoir temperatures. The degree of chemical equilibrium between groundwater solutes and reservoir rock is generally evaluated using the Na/1000-(K/100)-Mg^1/2^ ternary diagram^22^, from which the applicability of cation geothermometers can be assessed. In Fig. 3, except for sample DGJ-17, which plots in the full equilibrium region A, the compositions of most geothermal water samples all plot within the partial equilibrium region B. All the stream water samples plot compositionally near the Mg apex (region C) as immature waters. Partially and completely equilibrated waters can produce reliable reservoir temperatures from cation geothermometers^24^. The Semi spring has the highest reservoir temperature (~260 °C) followed by the Kawu spring (~250 °C) and the Dagejia spring (~220 °C) (Table S4).

Water-rock reactions in deep reservoirs are controlled by numerous factors including mineral composition, temperature, pressure, and fracture spacing. In general, the dissolution rate of a mineral is given by the equation:

1 $$\frac{d{c}_{i}}{dt}=\frac{{A}_{\theta }}{V}{\nu }_{\theta }{k}_{\theta }$$

assuming heterogeneous kinetics^25^. In this equation, *k*_*θ*_ is the dissolution rate constant of species *i* from mineral *θ*, *A*_*θ*_ is the surface area of the mineral, *V* is the volume of solution surrounding the mineral, ν_*θ*_ is the stoichiometric content of substance *i* in mineral *θ* and *C*_*i*_ is the concentration of species *i*. The temperature dependence of *k*_*θ*_ follows the Arrhenius equation:

2 $${k}_{\theta }=A{e}^{-{E}_{a}/RT}\cdot {({a}_{{H}^{+}})}^{{n}_{\theta }}$$

where *a*_*H+*_ is the activity of hydrogen ions; *n*_*θ*_ has values usually in the range of 0–1; *E*_*a*_ is the apparent activation energy of the dissolution reaction; *R* is the gas constant (8.314 J·mol^−1^·K^−1^) and T is the absolute temperature (K).

**Figure 4:**
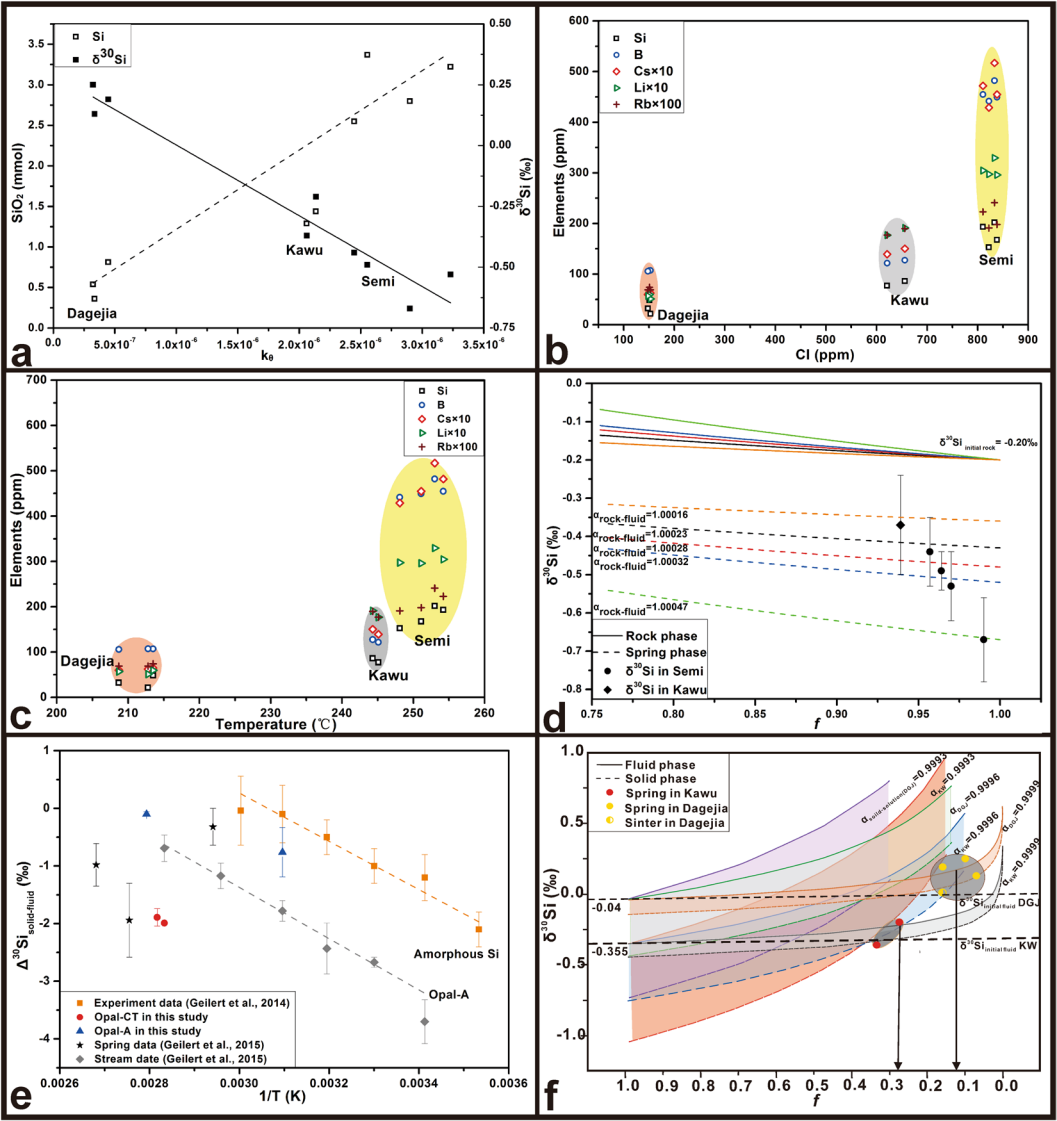
(**a**) Correlations of soluble silicon concentration and δ^30^Si with the dissolution rate constant *k*_*θ*_. (**b**) Correlations of B and RAM concentrations with Cl contents in spring water. (**c**) Correlations of B and RAM concentrations with reservoir temperature. (**d**) δ^30^Si_altered rock_ and δ^30^Si_fluid_ vs. *f* (fraction of silicon remaining in the altered rocks) for different α_rock-fluid_ values during water-rock interaction. (**e**) Correlations of Δ^30^Si_precipitate-solution_ vs. 1/T among different sinter minerals from this study and from previous studies^13,40^. (**f**) The variation of δ^30^Si_sinter_ and δ^30^Si_fluid_ with *f* (fraction of silicon remaining the solution) during the sinter precipitation for different α_sinter-solution_ values (the solid circle and half-solid circle represent the spring samples and the sinter samples, respectively).

From T and pH in each spring sites (Tables S1, S4), the rate constants of silicate mineral dissolution were derived by observing that the majority of *E*_*a*_ values fall within the range of −40 to −80 kJ/mol (a mean ca. −60 kJ/mol) and *n*_*θ*_ was taken to be −0.28, the value for feldspar at pH > 6^25^. As shown in Table 1, water-rock interaction was fastest at the Semi site; the rates of water-rock interaction (*k*_*θ*_) in the Semi reservoir are 1.3 and 7.6 times greater than those at Kawu and Dagejia reservoirs, respectively. Strong correlations of the concentrations of soluble silica with *k*_*θ*_ (an R^2^ value of 0.83) and of δ^30^Si with *k*_*θ*_ (an R^2^ value of 0.94) (Fig. 4a) are observed (Fig. 4a). The first correlation reflects effective leaching of silicate minerals due to elevated rates of water-rock interaction (*k*_*θ*_) in the reservoirs, which is also reflected in the strong positive correlations of B, Li, Rb and Cs concentrations with the reservoir temperature (Fig. 4c). The second correlation implies a direct response of δ^30^Si in the reservoirs to the kinetics of water-rock interaction, which is discussed below.

**Table 1:** Rate constants of water-rock interaction in the spring reservoirs.

Spring sites	Sample No	Reservoir temperature (°C)	pH	Rate constant (*k*_*θ*_, m^−2^·s^−1^)
Dagejia	DGJ-17	213.5	8.0	4.47 × 10^−7^
	DGJ-12	212.8	7.6	3.35 × 10^−7^
	DGJ-7	208.7	7.8	3.23 × 10^−7^
Kawu	KW-3-6	244.3	8.6	2.14 × 10^−6^
	KW-3-12	245.1	8.5	2.06 × 10^−6^
Semi	SM-38	253.0	8.4	2.55 × 10^−6^
	SM-39	254.2	8.7	3.23 × 10^−6^
	SM-40	248.1	8.6	2.45 × 10^−6^
	SM-41	251.1	8.7	2.90 × 10^−6^

As shown in Fig. S1, the reported δ^18^O and δD values of geothermal water from the Kawu, Dagejia, and Semi sites^16,26,27^, together with data for other geothermal waters in the region^1,18^, plot to the right of both the Global Meteoric Water Line (GMWL, δD = 8 × δ^18^O + 10) and a local meteoric water line for 5950 m above sea level (masl) (δD = 7.8 × δ^18^O + 8.7)^26^, indicating shifts in δ^18^O as a result of high-temperature water-rock interaction. The δ^18^O shift for the Semi site is larger than those for the Kawu and Dagejia sites, consistent with the Semi site having the highest reservoir temperature. All oxygen and hydrogen isotopic values of the geothermal waters plotted in Fig. S1 differ isotopically from those of 1990s rainwater from the Yarlung-Zangpo Gorge at 2450–4675 masl^27^ and from ice formed since AD 1864 on nearby Mt. Noijin Kangsang at 5950 masl^26^, consistent with deep circulation of ancient recharge^28^.

At Semi, the annual precipitation and evaporation are 318.5 mm and 2553.0 mm respectively, and at Dagejia, 192.6 mm and 2269.1 mm respectively^1^. The intense evaporation exerts less influence on the hydrochemical properties of up-welling spring water than stream water. At the Semi and Dagejia sites, the Cl/Br molar ratios exceed 1200, indicating an evaporite origin for Cl, whereas at the Kawu site, the Cl/Br ratios are lower (358–378, Table S1), possibly indicating a magmatic source. The Na/Cl molar ratios are close to unity at the Semi site, consistent with dissolution of halite as the dominant source of NaCl, which is supported by the presence of evaporites within Late Tertiary sandstone and conglomerate. In addition, the concentrations B, Cs and RAM increase with the Cl content (Fig. 4b), suggesting that elevated Cl^−^ concentrations may promote the leaching of RAM and boron from silicate minerals. On the other hand, this effect may not be separated from that of the temperature and pH in the present data set. The absence of a correlation of Cl/Br ratios with the enrichment of RAM and boron, however, indicates that the enrichment is independent of the source of chlorine.

It is noteworthy that the RAM and boron concentrations in the Kawu site are obviously lower than expected from water-rock interaction at inferred values of parameters such as T and pH (Fig. 4b,c). This could signal the influence of factors on the kinetics of water-rock interaction, such as mineral composition, fracture spacing (i.e., fracture width) and dislocation density of the host rocks, the viscosity of the fluid, and the hydrostatic pressure.

### Silicon isotope fractionation during water-rock interaction in deep reservoirs

In Fig. 2, the δ^30^Si values of the Dagejia geothermal water (+0.13 to +0.28‰) exhibit within the range of other geothermal waters (−0.4 to +0.7‰), e.g., Jiaodong, China^29^, Tenchong, China^30,31^, Yellowstone, USA^10^, mid-ocean ridge hydrothermal water^32^, and Iceland^11–13^ (Fig. 2). In Na-Cl type geothermal water from our study area, negative δ^30^Si values (−0.37 to −0.21‰ for Kawu, −0.67 to −0.44‰ for Semi) are accompanied by high SiO_2_, Cl, B, and RAM concentrations (Fig. 4a). The δ^30^Si values at the Semi site are the lowest reported to date for any geothermal water (Fig. 2). A possible explanation for this is that water-rock interaction reached an unprecedented high intensity and led to extreme kinetic fractionation of Si isotopes, as discussed below.

Silicon isotope fractionation associated with water-rock interaction is likely to be governed by kinetic effects. An alternative interpretation is suggested by the observation that the isotope fractionation accompanying leaching of silicon from reservoir rock as a process approximating Rayleigh fractionation. As the bond strength of ^30^Si-O is stronger than that of ^28^Si-O in silicate minerals by Wu *et al*.^33^, lighter Si isotopes are released preferentially into the aqueous phase during water-rock interaction^34^, resulting in increase of δ^30^Si values in altered rock under more intensive water-rock interaction. If the silicon isotope fractionation remains constant, δ^30^Si values in coexisting water will decrease as the geothermal system evolves.

In order to compare observed δ^30^Si values in discharging water with those in the reservoir host rock, it is necessary to estimate the δ^30^Si of the unaltered reservoir rock, which is inaccessible to sampling. This estimate was made using two approaches. In the first, we calculated δ^30^Si for a mixture of rock types based on the areal distribution of these rock-types around the springs, and in the second, we assumed that the δ^30^Si of the reservoir rock corresponded to that of the dominant rock-type in the region. As described above, the exposed rocks at each spring site are different. Granite is the major rock-type at the Kawu site, granite and siliciclastic sedimentary rocks dominate near the Dagejia site, and a more diverse assemblage (granite, ultramafic rocks, sedimentary rocks and metamorphic rocks) is present in the vicinity of the Semi site. The silicon isotopic compositions of the host rocks in geothermal reservoirs were estimated using the balance equation:

3 $${\delta }^{30}S{i}_{SR}={\delta }^{30}S{i}_{GR}\times w+{\delta }^{30}S{i}_{SER}\times x+{\delta }^{30}S{i}_{UR}\times y+{\delta }^{30}S{i}_{MR}\times z$$

where *w*, *x*, *y*, *z* corresponds to the molar proportions of granite, siliciclastic sedimentary rock, ultramafic rock and metamorphic rock, respectively, and *w* + *x* + *y* + *z* = 1; The subscripted δ^30^Si values are published values for these rock-types.

Based on the reported δ^30^Si values for granite (−0.5‰ to +0.3‰, average −0.2‰), siliciclastic sedimentary rock (−0.5‰ to +0.5‰, average −0.1‰), ultramafic rock (−0.7‰ to +0.4‰, with 90% in the range −0.4‰ to −0.2‰, average −0.3‰), and metamorphic rock (slate, −0.6‰ to +0.2‰, average −0.3‰; phyllite, −0.4‰ to 0.0‰, average −0.2‰)^35^, the weighted δ^30^Si_SR_ for the three sites has a value of −0.21‰ for a 90% statistical probability derived by iterative calculation for the study area (Fig. S2). The second approach makes use of the observation that lithology in the Indus Yarlung Zangbo Suture Zone in the Lhasa Terrane was dominated by the Mesozoic and Cenozoic granitic rocks^36^ and subvolcanic felsic porphyries^37^. The δ^30^Si values of granitic rocks vary in the range −0.5‰ to +0.3‰ (the average and median values are −0.2‰), whereas those of porphyries vary in the range −0.3‰ to +0.4‰ (the average and median values are −0.1‰ and −0.2‰), repectively^35^. This approach yielded a δ^30^Si value for the reservoir rocks of −0.20‰, which is almost identical to that estimated from the silicon isotopic compositions of the rocks exposed in the vicinity of the three hot spring sites. Considering the very high flow rates of the Semi, Degejia and Kawu hot springs (9.5 × 10^4^ m^3^·d^−1^, 7.9 × 10^6^ m^3^·d^−1^ and 6.3 × 10^5^ m^3^·d^−1^)^1^, the silicon isotope fractionation due to diffusion of hydrated silicon species should be negligible, although a fractionation factor ^30/28^α is estimated to be 0.998. This was estimated from the empirical inverse power relation proposed by Richter *et al*.^38^ (Eq. 4), might apply in static situations.

4 $${}^{30/28}\alpha =\frac{{D}_{30}}{{D}_{28}}\propto {(\frac{{m}_{28}}{{m}_{30}})}^{\beta }$$

where *D* is the diffusion coefficients of the silicon isotopes, *m* is the mass of the light or heavy isotope ignoring any water of hydration and *β* is the correction factor for monovalent ions, which varies from 0.01 to 0.06 (we used a value of 0.03^39^).

As a geothermal reservoir is an open system, the isotope fractionation accompanying the continuous leaching of silicon from silicate minerals can be treated as a fractional distillation under equilibrium conditions, described by the Rayleigh equation. From Fig. 4d, it is evident that the continuous leaching of silicon from silicate minerals during fluid circulation leads to simultaneous decreases of δ^30^Si in the spring water and increases of δ^30^Si in the altered rocks. According to the leaching-fractionation model, values of δ^30^Si_spring_ < −0.2‰ (as at the Semi and Kawu sites) represent early alteration, whereas values of δ^30^Si_spring_ > −0.2‰ reflect an advanced stage of alteration because of the higher δ^30^Si of the altered host rocks (as at the Dagejia site). In this interpretation, the RAM and boron are mainly transferred into the earliest, hottest geothermal water, which also has the highest Cl content. A silicon isotope fractionation factor, Δ^30^Si_solution-rocks_ of −0.16 to −0.47‰ was roughly estimated for the kinetic fractionation between rock and water in the Semi and Kawu sites.

### Silicon isotope fractionation during sinter precipitation

Silicon isotope fractionation during low-temperature precipitation of silicate minerals depends heavily on system parameters such as the precipitation rate and the solution chemistry. At low-temperature conditions (e.g., in groundwater and geothermal surface water), the minerals are enriched in ^28^Si, resulting in δ^30^Si values as low as −5.4‰ and −4.0‰, respectively^13,14,40,41^. Consequently, the residual solutions are enriched in ^30^Si through Rayleigh fractionation (Eqs 5–7).

5 $${{\rm{\delta }}}^{30}{{\rm{Si}}}_{{\rm{precipitate}}}={{\rm{\delta }}}^{30}{{\rm{Si}}}_{{\rm{solution}}}^{{\rm{i}}}+{{\rm{\Delta }}}^{30}{{\rm{Si}}}_{{\rm{precipitate}}-{\rm{solution}}}({\rm{1}}+\,\mathrm{ln}\,f)$$

6 $$f={C}_{{\rm{solution}}}/{C}_{{\rm{initial}}{\rm{solution}}}$$

7 $${{\rm{\Delta }}}^{30}{{\rm{Si}}}_{{\rm{precipitate}}-{\rm{solution}}}=1000{\mathrm{ln}{\rm{\alpha }}}_{{\rm{precipitate}}-{\rm{solution}}}={\delta }^{30}{{\rm{Si}}}_{{\rm{precipitate}}}-{\delta }^{{\rm{30}}}{{\rm{Si}}}_{{\rm{solution}}}$$

where the δ^30^Si_precipitate_ and δ^30^Si^i^_solution_ denote the Si isotope compositions of SiO_2_ precipitates and the initial hydrothermal solution, respectively. The term Δ^30^Si_solid-solution_ (i.e. 1000lnα_solid-solution_) denotes the fractionation between the hydrothermal fluid and the precipitated SiO_2_, and *f* denotes the fraction of silica remaining in solution, as expressed by Eq. 6. The terms *C*_solution_ and *C*_initial solution_ are the silicon concentrations in geothermal water venting at the surface and in hydrothermal fluid at high temperatures in the deep reservoir, respectively.

Assuming equilibrium, the value of *C*_initial solution_ can be determined from the equilibrium constant for the reaction SiO_2 (s)_ + 2H_2_O ↔ H_4_SiO_4_ at the typical reservoir temperature using Eq. 8 ^42^. The δ^30^Si value of the initial solution was determined from Eq. 9.

8 $$\mathrm{log}\,{\rm{K}}=\,-\,{\rm{8.476}}-{\rm{485}}{{\rm{.24T}}}^{-1}-2.268\times {10}^{-{\rm{6}}}{{\rm{T}}}^{2}+3.068{\rm{logT}}$$

9 $${{\rm{\delta }}}^{30}{{\rm{Si}}}_{{\rm{solution}}}^{{\rm{i}}}={{\rm{\delta }}}^{30}{{\rm{Si}}}_{{\rm{solution}}}-{{\rm{\Delta }}}^{30}{{\rm{Si}}}_{\mathrm{precipitate}-\mathrm{solution}}\,\mathrm{ln}\,({{\rm{C}}}_{{\rm{solution}}}/{{\rm{C}}}_{{\rm{initial}}{\rm{solution}}})$$

The silicon isotope fractionation factor, *α*, is related to temperature T (K) by the equation of $\alpha ={e}^{-{\rm{\Delta }}E/RT}$^30^, which yields a linear relationship between Δ^30^Si_solid-solution_ and 1/T for each sinter mineral (Fig. 4e). At constant temperature, α_solid-solution_ decreases with decreasing crystallinity in the sequence of α_opal CT-solution_ > α_opal A-solution_ > α_amorphous silica-solution_, reflecting differences in the activation energy, −52.1, −41.6 and −36.9 kJ·mol^−1^, of their respective formation reactions. As Opal-A was the first stable phase to crystallize from amorphous silica and was subsequently transformed into Opal-CT^43^, it follows that the δ^30^Si values of Opal-A provide the best basis for calculating δ^30^Si_solution_^i^ of the hydrothermal fluid (Table S3).

In geothermal systems, the rate of silica precipitation imposes a kinetic effect on silicon isotope fractionation between sinter and solution. This rate was calculated using Eqs 10–12 ^13^.

10 $${\rm{rate}}=\frac{d[{H}_{4}Si{O}_{4}]}{dt}=k(\frac{A}{M})(1-\frac{Q}{K})$$

11 $$Q={a}_{{H}_{4}Si{O}_{4}}/{a}_{Si{O}_{2}}\times {a}_{Si{O}_{2}}^{2}$$

12 $$\mathrm{log}\,k=\,-\,0.369-7.890\times {10}^{-4}{\rm{T}}-3438/{\rm{T}}$$

where *Q* and *K* are the activity product and the equilibrium constant of the reaction H_4_SiO_4_ ↔ SiO_2 (s)_ + 2H_2_O, respectively (Eqs 8, 10). *k* is the rate constant, which depends on the temperature (Eqs 10, 12), A is the interfacial area and M is the mass of water.

The A/M ratio is one of key parameters for estimating the precipitation kinetics of amorphous silica. It was estimated assuming a flat open stream channel with a uniformly shallow water depth where there are no borders because of the shallow depth relative to surface area, and a constant specific volume of water (i.e., independent of the temperature variation). The resulting A/M ratio will be 0.1 if the units are expressed in meters and kilograms for 1 cm of water depth^10,13^. For this geometry, the precipitation rates are 3.9 × 10^−12^ to 5.2 × 10^−12^ mol·L^−1^·s^−1^ at the Dagejia site, 2.2 × 10^−12^ to 3.6 × 10^−12^ mol·L^−1^·s^−1^ at the Semi site and 3.9 × 10^−12^ to 4.1 × 10^−12^ mol·L^−1^·s^−1^ at the Kawu site (Table S5, Fig. S3). These rates are higher than the rates of 2.0 × 10^−13^ to 4.7 × 10^−13^ mol·L^−1^·s^−1^ for the Geysir Konungshver springs^13^. During sinter precipitation, the first step is the formation of SiO_2_·H_2_O gel as a result of the sharp drop in the pressure and temperature of the spring water as it vents at the surface. As the solubility of silicic acid increases with increasing pH, the higher pH of the Semi and Kawu springs compared to the Dagejia spring inhibited sinter precipitation. In addition, the higher contents of alkali and/or alkaline earth metals tends to neutralize the negatively charged metastable SiO_2_ particles and promotes the coagulation of SiO_2_·H_2_O gel. The capacity to enhance coagulation is related to radius and atomic mass in the sequence Cs > Rb > K > Na > Li^43^. The high RAM concentrations at the study sites lead to higher precipitation rates than in other geothermal systems, such as those in Iceland and Cistern in the Yellowstone National Park, USA^10,44^.

The isotopic fractionation during sinter formation was evaluated by comparing the predicted variations of δ^30^Si_solid_ and δ^30^Si_fluid_ with *f* for different α_solid-solution_ values to the average calculated δ^30^Si^i^_solution_ values of −0.055‰ for the Dagejia site and −0.34‰ (KW-3-6) for the Kawu site (Fig. 4f). The observed distribution of δ^30^Si_sinter_ and δ^30^Si_spring_ values corresponds to an α_solid-solution_ value of ~0.9998–0.9999 consistently, showing that rapid coagulation of silica gel from saline solutions precludes isotope fractionation. This explains why silicon isotope fractionation during the sinter precipitation in Tibet is less than that in low-salinity geothermal systems in Iceland (α_solid-solution_ = 0.9993)^13^ and in experimental abiotic silica precipitation (α_solid-solution_ = 0.9996)^30^. Accordingly, the percentages of silicon removed from solution by precipitation at Kawu and Dagejia are 78% and 88%, respectively (Fig. 4f), consistent with the greater amount of siliceous sinter exposed at the Dagejia site.

### Integrated kinetic effects on silicon isotope signatures

Silicon circulation in geothermal systems is a dynamic process, relating the source where silicon is leached from silicate minerals to the sinks where sinter is precipitated around geothermal springs. The rates of water-rock interaction and sinter precipitation follow the order of Semi > Kawu > Dagejia and Dagejia > Kawu > Semi respectively. The very high dissolution rate and very low precipitation rate at Semi explain why this spring has the highest concentration of soluble Si and the most negative δ^30^Si. By analogy, the very low precipitation rate and very high dissolution rate at Dagejia explain the very low concentration of soluble Si and the positive δ^30^Si signature of that spring, which are similar to those of geothermal springs elsewhere. The intermediate dissolution and precipitation rates at the Kawu site lead to δ^30^Si values closer to the host rocks. In general, silicon isotope fractionation during sinter precipitation (Δ^30^Si_precipitate-solution_ is <−0.1‰) is less significant than that in water-rock interaction (Δ^30^Si_solution-rocks_, at least as high as −0.47‰). This enables the δ^30^Si signatures of spring waters to be used to evaluate the impact of fluid temperature on the intensity of water-rock interaction in geothermal reservoirs.

## Discussion

**Figure 5:**
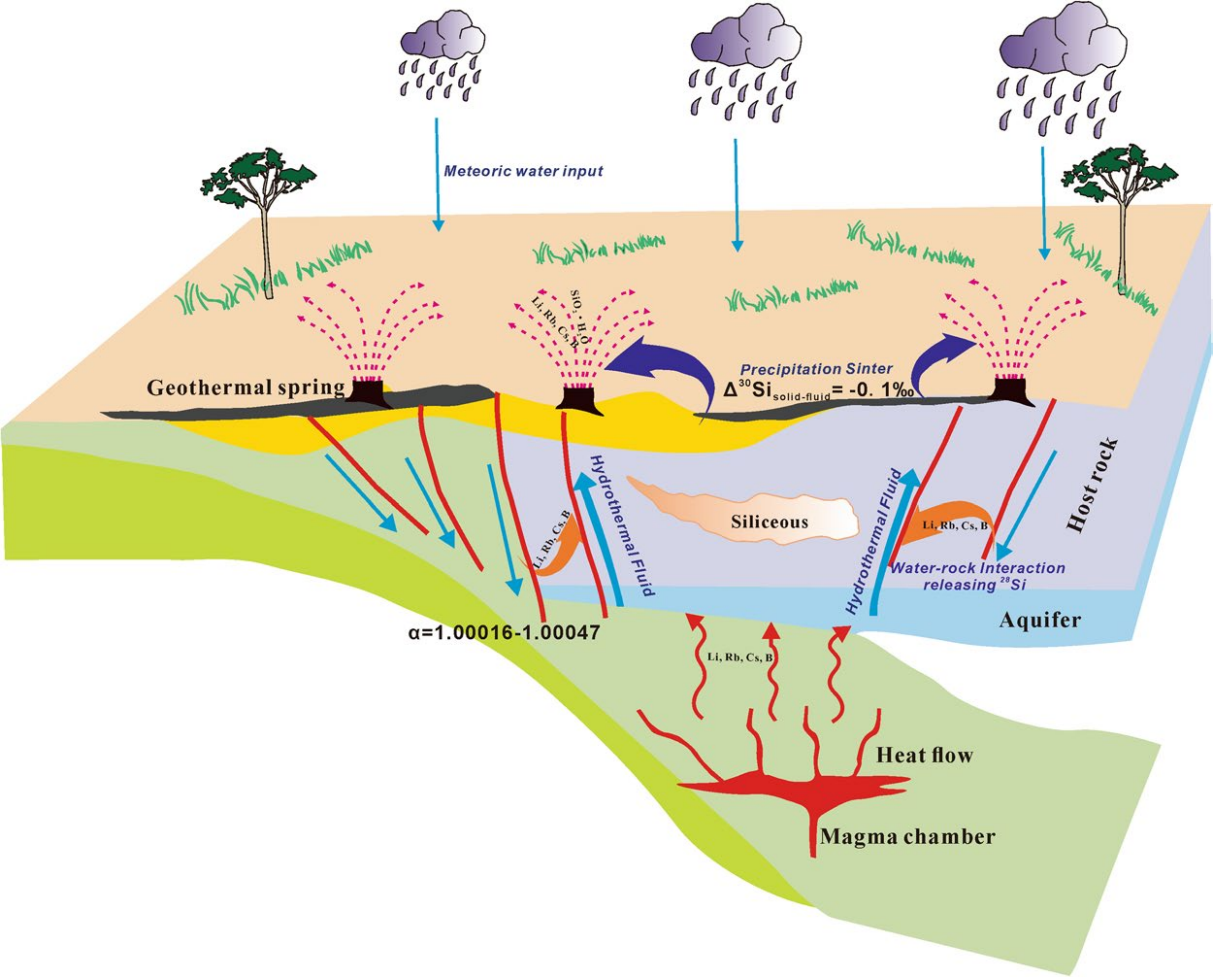
A conceptual model of geothermal circulation, showing silicon cycling and associated silicon isotope fractionation.

The mantle-derived Gangdese magmatic arc, with which geothermal activity in south Tibet is associated, was emplaced after the India-Asia plate collision and later underwent Oligocene metamorphic and anatectic reworking^2^. The partial melts from the enriched mantle wedge redistributed incompatible elements in the underthrust terrane, including the geothermal reservoir rocks of the study area. A conceptual model describing silicon cycling and isotope fractionation during circulation is given in Fig. 5. In the Lhasa Terrane, persistent magmatism provides continuous heat-flow that drives geothermal circulation. The δ^11^B and ^3^He signatures in the geothermal waters^3,4^ are consistent a role for magma degassing in concentrating boron, and probably also RAM. Secondary, strong water-rock interactions between the geothermal fluid and granitic rocks appears to lead to the enrichment of RAM in the geothermal springs. The evidence presented above leads to the following general observations for the three geothermal systems considered in this study: (i) enrichment of the RAM correlates with the high Cl^−^ content of the geothermal water and high geothermal residence temperature, factors that are essential in promoting mineral alteration and the leaching of RAM from the host rocks; (ii) the silicon isotope fractionation during precipitation of amorphous sinter is less significant than that during water-rock interaction, which allows the δ^30^Si signatures in the springs to be used to evaluate the intensity of water-rock interaction; (iii) the association of the highest RAM concentrations with the lowest δ^30^Si and the most extreme δ^18^O shift are consistent with the hypothesis that intense water-rock interaction favors accumulation of RAM in geothermal fluids; (iv) assuming that the evolution of δ^30^Si in the wall-rocks was due to Rayleigh fractionation, it follows that RAM and boron were removed early in the alteration; (v) the entire δ^18^O dataset^26,27,45^ suggests that the geothermal waters of this study most likely recharged thousands of years ago, allowing a large amount of time for water-rock reaction; (vi) sinter formation can further concentrate RAM.

The δ^30^Si values and concentration of dissolved Si in the geothermal waters of the study area have provided a new perspective on the kinetics of silicon migration between silicate minerals and geothermal water, which has allowed us to show that the δ^30^Si signatures of springs can be used to evaluate the intensity of water-rock interaction and controls on the RAM enrichment. The processes of water-rock interaction and silica precipitation, however, are more complex than envisaged. Firstly, the factors that control water-rock interaction extend far beyond equilibrium solution chemistry, and likely to include the mineralogy and mechanical properties of the reservoir rocks (e.g., fracture spacing, permeability, and density etc.), and the physical properties of the fluid (e.g., viscosity, pressure etc.). Secondly, the precipitation of silica appears to be a non-equilibrium process, resulting in deposits exceeding the size predicted by equilibrium silica solubility. This departure from equilibrium is likely due to the fact that the precipitation begins with the nucleation of colloidal amorphous silica particles. In addition to the concentration of dissolved Si, temperature, pH and other cations (e.g., alkali and/or alkaline earth metals, Al^3+^) also affect the precipitation of silica and its subsequent transformation. These observations need to be addressed in future studies.

## Methods

Geothermal water and siliceous sinter were collected from three geothermal fields (Dagejia, Semi and Kawu) in southern Tibet, along the Indus-Yarlung Zangbo River Suture Zone. River water samples were collected from the Yalu Tsangpo River in the Semi area and the Changmaqu River in the Dagejia area. Silicon isotopes and elemental concentrations of rock and water samples were measured in the State Key Laboratory for Mineral Deposits Research, Nanjing University. Silicon isotope compositions of geological standard materials were measured in this study and compared to their recommended values (Table 2). A full description of the methods and analytical precisions (given as 2σ), including elemental and physicochemical parameters of aqueous and solid samples, crystal structure characteristics and silicon isotope analysis, is given in the following sections.

**Table 2:** Silicon isotope compositions in the standard geological materials.

Sample No	Mineral types	*δ*^30^Si _measured_ (2σ)	*δ*^30^Si _given_ (2σ)	Reference
GBW-04421	Quartz	+0.01 ± 0.02‰	−0.02 ± 0.10‰	^49^
GBW-04422	Quartz	−2.79 ± 0.05‰	−2.68 ± 0.10‰	^49^
B-5	Porphyritic basalt	−0.89 ± 0.06‰	−1.0~−0.3‰	^31, 33^
B-6	Obsidian	−0.24 ± 0.02‰	−0.4~0.4‰	^31, 33^
GSR-3	Basalt	−0.68 ± 0.08‰	−1.0~−0.3‰	^31, 33^

### Elemental and physicochemical parameters analysis of aqueous samples

Physicochemical parameters of aqueous samples (e.g. temperature, pH, electrical conductivity (EC) and total dissolved solids (TDS) were measured immediately at the sampling sites with a calibrated portable multi-parameter analyzer (HQ40d, HACH America). Aqueous samples were stored in acid-washed, high-density polyethylene sampling bottles after being filtered through cellulose filters (0.45 µm), and acidified with 1 M HNO_3_, to avoid any invisible precipitation in the case of cation analysis. Cation concentrations were analyzed by Inductively Coupled Plasma Optical Emission Spectrometry (ICP-OES, Agilent-710) with an analytical reproducibility of ±3% in the Public Technical Service Center of Nanjing Institute of Geology and Palaeontology, Chinese Academy of Sciences. Anion concentrations were measured on an ion chromatograph (IC, type Thermo Fisher ICS-900) with an analytical reproducibility of ±3%, and trace elements were analyzed by Inductively Coupled Plasma Mass Spectrometry (ICP-MS, Element II, Thermo Fisher Finnigan) with an analytical reproducibility of ±5% at the State Key Laboratory for Mineral Deposits Research, Nanjing University.

### Major and trace element analysis of solid samples

The major and trace element contents of the opal samples were measured by XRF and ICP-MS at the State Key Laboratory for Mineral Deposits Research, Nanjing University. The solid samples were crushed and powdered to 200-mesh using an agate mill. Major element compositions were determined by X-ray fluorescence spectroscopy (XRF) using an AXIOS Mineral Spectrometer, with an analytical uncertainty of <±5% (2σ), following the procedure of Norrish and Hutton^46^. Trace element concentrations were analyzed on an ICP-MS (Finnigan Element II). About 50 mg of powdered sample was dissolved in high-pressure Teflon containers using a HF + HNO_3_ acid mixture acid for 48 h at approximately 160 °C. Rh was used as an internal standard to monitor signal drift during ICP-MS measurement. The analytical precision was better than ±5% for most trace and rare earth elements. Details of the analytical procedures are described by Gao *et al*.^47^.

### XRD analyses of solid samples

Samples for X-ray diffraction (XRD) (Bede-D1) with CuKα radiation (X’ TRA) were dried and ground to a grain size of about 200 µm. The analyses were performed over a 2θ interval between 3° and 51°, using a step of 0.02° and an integration time of 0.24 s/step. The XRD spectra were analyzed by Jade 5.0 software to identify the minerals in the solid samples.

### Dissolution of solid samples for Si isotope measurement

Standard samples were fused with K_2_CO_3_ (SP, Aldrich-Sigma, 99.99%) in platinum crucibles for 150 min at 950 °C in a muffle furnace. After cooling in air, the fusion cake was dissolved in Milli-Q water (resistivity, 18.2 MΩ·cm) and acidified with 3 M HNO_3_. The sample solution was diluted to about 60 ppm SiO_2_ and the solution pH was adjusted to ~7.0 with HNO_3_ in order to prevent polymerization.

### Separation and purification of silicon

Both samples and silicon isotope standard materials (including the bracketing standard) were purified using single column chemistry. The poly-prep 10 mL column (BioRad, USA) is filled with 1.5 mL DOWEX AG 50W-X8 (200–400 mesh) cation exchange resin. The resin was regenerated before each sample loading by sequential washing with 3 mL 3 M HCl, 3 mL 6 M HCl, 3 mL 7 M HNO_3_, 3 mL10M HCl, 3 mL 6 M HCl, 3 mL 3 M HCl and Milli-Q water washing to neutral pH. At low pH (pH < 8), dissolved Si in the form of non-ionic monosilicic acid Si(OH)_4_ or the anionic species H_3_SiO_4_^−^ that is not retained by the resin. For each sample, an aliquot of solution containing about 60 μg Si was loaded onto a column. Samples were eluted in about 10 mL of Milli-Q water and, after purification, were diluted with Milli-Q water to 15 mL, which contains about 4 ppm Si for silicon isotope analysis. The typical overall column recovery of Si was greater than 95%.

### Silicon isotope analysis

Silicon isotope were analyzed using the method of Georg *et al*.^48^ with a slight modification. The effects of other major anionic species (e.g. SO_4_^2−^, Cl^−^) on silicon isotope values were evaluated in this work, and are described in detail in the Supplementary Information. Measurements were made on a Neptune Plus MC-ICP-MS (Thermo Fisher Finnigan, Germany) with an ESI PFA 50 μL/min nebulizer in a quartz cyclonic spray chamber. The ^28^Si^+^, ^29^Si^+^, and ^30^Si^+^ ions were collected by Faraday cups L3, central, and H3. Potential isobaric inference from ^14^N^16^O^+^ (m/z = 29.997989) on the ^30^Si^+^ion (m/z = 29.97377) was eliminated by operating with the medium resolution mode of ∼5000. The silicon content in both the sample solution and standard material NIST 28 was kept at ∼4 ppm to maintain a ^28^Si^+^ signal of ∼9.0 V in wet plasma mode and the Si signal was washed down to <30 mV. Instrumental mass bias was corrected by a sample-standard bracketing procedure (SSB). Results are expressed in delta notation (δ^30^Si) as the per mil (‰) deviation from the standard material NBS 28 (Eq. 13).

13 $${\delta }^{30}Si=\{\frac{{{(}^{30}Si{/}^{28}Si)}_{sample}}{0.5\times [{{(}^{30}Si{/}^{28}Si)}_{std-1}+{{(}^{30}Si{/}^{28}Si)}_{std-2}]}-1\}\times 1000$$

The abbreviations std-1 and std-2 refer to the standard material of NBS-28 measured before and after each sample, respectively. The international standard NBS-28 and the Chinese silicon isotope standard materials GBW-04421 and GBW-04422 (in quartz form) were prepared using the procedure described above. The silicon isotope compositions in the standard reference materials (GBW-04421, GBW-04422) values agree well with previous estimates (Table 2), ensuring accurate analysis of silicon isotopes in this study. All estimates of reproducibility described in this paper are from replicated measurements (n ≥ 4, 95% confidence limit). The long-term instrumental reproducibility, expressed as the standard deviations for isotopic reference material NBS 28 was ± 0.06‰ (n = 8, 2σ).

## Supplementary information

Final clean version of Supplementary Information
