# Supplementary material for: The origin of rare alkali metals in geothermal fluids of southern Tibet, China: A silicon isotope perspective
Source: Sci Rep. 2019 May 27;9:7918. doi: 10.1038/s41598-019-44249-5 (PMC6536511; doi:10.1038/s41598-019-44249-5)
Supplement: Supplementary file 1 — Final clean version of Supplementary Information [file 41598_2019_44249_MOESM1_ESM.docx]

**Supplementary information**

**The origin of rare alkali metals in geothermal fluids of southern Tibet, China: A silicon isotope perspective**

Wei Wang^1^, Hai-Zhen Wei ^2*^, Shao-Yong Jiang ^1,2*^, Hong-Bing Tan ^3*^, C. J. Eastoe ^4^,

Anthony E. Williams-Jones ^5^, Simon V. Hohl ^2^, He-Pin Wu ^2^

*^1^ State Key Laboratory of Geological Processes and Mineral Resources, School of Earth Resources, China University of Geosciences, Wuhan 430074, PR China*

*^2^ State Key Laboratory for Mineral Deposits Research, School of Earth Sciences and Engineering, Nanjing University, Nanjing 210023, PR China*

*^3^ School of Earth Sciences and Engineering, Hohai University, Nanjing 210098, PR China*

*^4^Department of Geosciences, University of Arizona, Tucson, Arizona 85721, United States*
^5^ *Department of Earth and Planetary Sciences, McGill University, Montreal H3A 0E8, Canada*

*: [haizhenwei@nju.edu.cn](mailto:haizhenwei@nju.edu.cn); [shyjiang@nju.edu.cn](mailto:shyjiang@nju.edu.cn); tan815@sina.com

**Results**

*Elemental concentrations in springs and streams*

**Table S1.** Chemical composition of geothermal hot spring and streams

| **Sample no.** | **Location** | **Distance to**  **Spring (m)** | **pH** | **Ion Concentrations (ppm)** | | | | | | | | | | | | |
| --- | --- | --- | --- | --- | --- | --- | --- | --- | --- | --- | --- | --- | --- | --- | --- | --- |
|  |  |  |  | **HBO_2_** | **Na** | **Mg** | **Al** | **SiO_2_** | **K** | **Ca** | **Fe** | **Li** | **Rb** | **Cs** | **Cl** | **Br** |
| DGJ-17 | Spring |  | 8.0 | 107 | 345 | BD | 0.27 | 48.67 | 33.11 | 1.46 | BD | 5.97 | 0.74 | 6.48 | 151 | 0.23 |
| DGJ-12 | Spring | 0 | 7.6 | 107 | 338 | BD | 0.20 | 21.58 | 32.14 | 3.41 | 0.06 | 5.11 | 0.69 | 6.23 | 153 | 0.22 |
| DGJ-7 | Spring |  | 7.8 | 106 | 333 | BD | 0.10 | 32.29 | 30.17 | 3.51 | BD | 5.69 | 0.69 | 6.00 | 148 | 0.23 |
| DGJ-15 | stream | -1000 |  | 6.12 | 16.13 | 2.95 | BD | 2.30 | 1.60 | 27.43 | BD | 0.12 | 0.12 | 0.12 | 6.15 | 0.00 |
| DGJ-5 | stream | 10 |  | 39.75 | 95.51 | 1.52 | BD | 36.52 | 10.10 | 16.30 | BD | 1.83 | 0.29 | 2.19 | 59.0 | 0.13 |
| DGJ-1 | stream | 200 |  | 42.03 | 93.92 | 2.26 | BD | 19.48 | 7.68 | 19.25 | 0.05 | 1.73 | 0.27 | 1.75 | 43.2 | 0.06 |
| DGJ-20 | stream | 1000 |  | 29.78 | 82.83 | 2.39 | BD | 25.92 | 7.29 | 20.98 | 0.07 | 1.48 | 0.23 | 1.48 | 38.7 | 0.07 |
| KW-3-6 | Spring |  | 8.6 | 127 | 556 | 0.10 | 0.18 | 86.28 | 75.19 | 5.53 | 0.07 | 19.12 | 1.90 | 15.04 | 656 | 4.13 |
| KW-3-12 | Spring (deep) |  | 8.5 | 122 | 503 | BD | BD | 77.28 | 68.59 | 2.70 | BD | 17.69 | 1.77 | 13.92 | 621 | 3.70 |
| SM-7 | Mountain spring water | -700 |  | 2.68 | 3.43 | 3.17 | BD | 5.75 | 1.51 | 60.52 | 0.03 | 0.05 | 0.11 | 0.12 | 0.12 | 0.05 |
| SM-14 | Stream | -600 |  | 3.62 | 10.28 | 7.08 | BD | 0.32 | 1.77 | 49.00 | 0.06 | 0.13 | 0.11 | 0.10 | 7.01 | 0.02 |
| SM-2 | Stream | 0 |  | 6.42 | 11.86 | 8.04 | 0.11 | 2.88 | BD | 55.93 | BD | 0.16 | 0.12 | 0.14 | 4.77 | 0.19 |
| SM-26 | Stream | 1000 |  | 3.69 | 12.28 | 7.80 | 0.00 | 0.84 | 1.51 | 53.17 | 0.07 | 0.16 | 0.11 | 0.16 | 6.15 | 0.00 |
| SM-38 | Spring | 0 | 8.4 | 482 | 753 | 0.80 | 0.00 | 202 | 111 | 34.51 | 0.10 | 32.95 | 2.41 | 51.70 | 833 | 2.01 |
| SM-39 | Spring | 400 | 8.7 | 455 | 691 | 1.44 | 0.58 | 193 | 104 | 16.33 | 1.52 | 30.45 | 2.23 | 48.19 | 810 | 1.91 |
| SM-40 | Spring | 600 | 8.6 | 442 | 620 | 0.25 | 0.00 | 153 | 87.27 | 8.20 | 0.07 | 27.93 | 1.91 | 42.90 | 822 | 1.81 |
| SM-41 | Spring | 800 | 8.7 | 449 | 678 | 0.25 | 0.15 | 168 | 98.38 | 4.59 | 0.07 | 29.69 | 1.98 | 45.48 | 838 | 1.84 |

BD: below the detection limit.

Note: For the Dagejia site, the negative number in the location column represents samples located to the south of the main spring (DGJ-12), and the positive number represents samples located to the north. The Semi springs are distributed along the Yarlung Zangbo river, and one of the most obvious landmarks is a bridge over the river (we took spring and stream samples under the bridge), which is considered to be the center point (0 m). The nagative number represents samples located to the west of the spring, and the positive number represents samples located to the east of the spring. Sample KW-3-12 (deep) at Kawu was taken directly from a drill pipe, which records the original composition of the deep reservoir fluid, avoiding the effect of precipitation close to the surface. Sample KW-3-6 was taken from the surface of the spring.

*Elemental concentrations in solid samples*

**Table S2.** Geochemical composition of siliceous sinters in study area

| Sample no | Mineralogy | Content of major elements  (wt. %) | | | | | | | | | | Content of trace elements  (ppm) | | |
| --- | --- | --- | --- | --- | --- | --- | --- | --- | --- | --- | --- | --- | --- | --- |
|  |  | SiO_2_ | TiO_2_ | Al_2_O_3_ | Fe_2_O_3_ | MnO | MgO | CaO | Na_2_O | K_2_O | P_2_O_5_ | Li | Rb | Cs |
| DGJ-8 | Opal-CT | 93.7 | 0.04 | 0.77 | BD | 0.01 | 0.14 | 0.74 | 0.41 | 0.29 | 0.02 | 16.50 | 64.63 | 2034 |
| DGJ-9 | Opal-A | 90.73 | 0.08 | 1.43 | 0.21 | 0.00 | 0.18 | 0.67 | 0.28 | 0.39 | 0.04 | 11.37 | 75.02 | 2324 |
| DGJ-10-1 | Opal-A | 89.7 | 0.02 | 2.53 | BD | 0.01 | 0.15 | 0.74 | 0.69 | 0.52 | 0.02 | 29.80 | 340 | 10662 |
| DGJ-11 | Opal-CT | 92.35 | 0.01 | 0.41 | BD | 0.01 | 0.14 | 0.67 | 0.50 | 0.27 | 0.03 | 20.12 | 69.28 | 2176 |

BD: below the detection limit.

*Silicon isotope compositions in spring and stream*

**Table S3.** Silicon isotope compositions of aqueous and solid samples

| **Location** | **Sample no.** | **Distance (m)** | **Type** | **δ^30^Si_measured_ (2σ, ‰)**  **(n=4)** | | **δ^30^Si_initial solution_ (‰)** | |
| --- | --- | --- | --- | --- | --- | --- | --- |
| **Aqueous samples in the Dagejia site** | | | | |  | |  |
|  | DGJ-7 |  | Spring | +0.25±0.03 | | 0.00 | |
|  | DGJ-12 | 0 | Spring | +0.13±0.03 | | -0.13 | |
|  | DGJ-17 |  | Spring | +0.19±0.04 | | 0.02 | |
|  | DGJ-15 | -1000 | Stream | -0.32±0.10 | |  | |
|  | DGJ-5 | 10 | Stream | +0.01±0.13 | |  | |
|  | DGJ-1 | 200 | Stream | -0.14±0.08 | |  | |
|  | DGJ-20 | 1000 | Stream | -0.79±0.10 | |  | |
| **Solid samples in the Dagejia site** | | | | | |  | |
|  | DGJ-8 |  | Opal-CT | -1.70±0.15 | |  | |
|  | DGJ-9 |  | Opal-A | -0.57±0.43 | |  | |
|  | DGJ-10-1 |  | Opal-A | +0.09±0.04 | |  | |
|  | DGJ-11 |  | Opal-CT | -1.80±0.03 | |  | |
| **Aqueous samples in the Semi site** | | | | | |  | |
|  | SM-38 | 0 | Spring | -0.49±0.05 | | -0.53 | |
|  | SM-39 | 400 | Spring | -0.53±0.09 | | -0.58 | |
|  | SM-40 | 600 | Spring | -0.44±0.09 | | -0.51 | |
|  | SM-41 | 800 | Spring | -0.67±0.11 | | -0.73 | |
|  | SM-2 | 0 | Stream | +0.10±0.12 | |  | |
|  | SM-26 | 1000 | Stream | +0.54±0.03 | |  | |
|  | SM-7 |  | Mountain spring water | +0.23±0.08 | |  | |
| **Aqueous samples in the Kawu site** | | | | | |  | |
|  | KW-3-6 |  | Spring | -0.21±0.11 | | -0.34 | |
|  | KW-3-12 |  | Spring | -0.37±0.13 | |  | |

Note: The negative number in the location column represents samples located to the south of the spring

δ^30^Si_initial solution_ was the silicon isotope value in the initial solution, which was calculated using Eq. 9.

*Reservoir temperatures reconstructed in the study area*

**Table S4.** Outflux temperatures of geothermal water measured at the sampling sites and reservoir temperature calculated by different geothermometers

| **Geothermometer (⁰C)** | **KW-3-6** | **KW-3-12** | **DGJ-7** | **DGJ-12** | **DGJ-17** | **SM-38** | **SM-39** | **SM-40** | **SM-41** |
| --- | --- | --- | --- | --- | --- | --- | --- | --- | --- |
| **T/⁰C_measured_** | 84.5 | 84.5 | 82.0 | 85.0 | 82.0 | 83.0 | 79.0 | 76.0 | 84.0 |
| **Quartz_no stream loss_** | 129.1 | 123.3 | 79.6 | 82. 3 | 83.5 | 180.7 | 177.6 | 162.4 | 168.3 |
| **Quartz_max stream loss_** | 125.9 | 121.0 | 83. 3 | 85.6 | 86.7 | 168.7 | 166.2 | 153.7 | 158.6 |
| **Chalcedony_no stream loss_** | 101.6 | 95.2 | 48.3 | 51.1 | 52.4 | 159.7 | 156.2 | 138.7 | 145.5 |
| **Chalcedony_max stream loss_** | 101.5 | 96.2 | 56.8 | 59.2 | 60.4 | 147.6 | 144.9 | 131.2 | 136.6 |
| **Na-K-Ca** | 236.1 | 242.6 | 204.3 | 207.8 | 217.6 | 228.4 | 235.7 | 236.8 | 247.8 |
| **Na-K** | 244.3 | 245.1 | 208.7 | 212.8 | 213.5 | 253.0 | 254.2 | 248.1 | 251.1 |

*Precipitation rate from geothermal water in study area*

**Table S5.** Precipitation rate from geothermal water in the study area

| **Location** | **Sample no.** | **Distance**  **(m)** | **Type** | **Soluble SiO_2_ content**  **(mol⋅L^-1^)** | **T**  **(^0^C)** | | ***k***  **(s^-1^)** | | **K** | | **Q/K** | **A/M** | **Rate**  **(mol⋅L^-1^⋅s^-1^)** |
| --- | --- | --- | --- | --- | --- | --- | --- | --- | --- | --- | --- | --- | --- |
| **Dagejia** | | | | |  |  | |  | |  |  |  | |
|  | DGJ-7 | -100 | Spring | 0.00081 | 82 | | 4.6×10^-11^ | | 0.00496 | | 0.16 | 0.1 | 3.9×10^-12^ |
|  | DGJ-12 | 0 | Spring | 0.00036 | 85 | | 5.6×10^-11^ | | 0.00517 | | 0.07 | 0.1 | 5.2×10^-12^ |
|  | DGJ-17 | 0 | Spring | 0.00054 | 82 | | 4.6×10^-11^ | | 0.00496 | | 0.11 | 0.1 | 4.1×10^-12^ |
|  | DGJ-15 | -1000 | Stream | 0.00004 | 19 | | 4.2×10^-13^ | | 0.00171 | | 0.02 | 0.1 | 4.1×10^-14^ |
|  | DGJ-5 | 10 | Stream | 0.00061 | 19 | | 4.2×10^-13^ | | 0.00171 | | 0.36 | 0.1 | 2.7×10^-14^ |
|  | DGJ-1 | 200 | Stream | 0.00032 | 15 | | 2.9×10^-13^ | | 0.00157 | | 0.21 | 0.1 | 2.3×10^-14^ |
|  | DGJ-20 | 1000 | Stream | 0.00043 | 12 | | 2.2×10^-13^ | | 0.00147 | | 0.29 | 0.1 | 1.6×10^-14^ |
| **Semi** | | | | |  | |  | |  | |  |  |  |
|  | SM-38 | 0 | Spring | 0.00186 | 83 | | 5.1×10^-11^ | | 0.00506 | | 0.37 | 0.1 | 3.2×10^-12^ |
|  | SM-39 | 400 | Spring | 0.00173 | 79 | | 3.8×10^-11^ | | 0.00474 | | 0.36 | 0.1 | 2.4×10^-12^ |
|  | SM-40 | 600 | Spring | 0.00145 | 76 | | 3.2×10^-11^ | | 0.00456 | | 0.32 | 0.1 | 2.2×10^-12^ |
|  | SM-41 | 800 | Spring | 0.00164 | 84 | | 5.2×10^-11^ | | 0.00510 | | 0.32 | 0.1 | 3.6×10^-12^ |
|  | SM-2 | 0 | Stream | 0.00000 | 10 | | 1.8×10^-13^ | | 0.00141 | | 0.00 | 0.1 | 1.8×10^-14^ |
|  | SM-26 | 1000 | Stream | 0.00003 | 14 | | 2.7×10^-13^ | | 0.00154 | | 0.02 | 0.1 | 2.6×10^-14^ |
|  | SM-7 |  | Mountain spring water | 0.00003 | 15 | | 2.9×10^-13^ | | 0.00157 | | 0.02 | 0.1 | 2.9×10^-14^ |
| **Kawu** | | | | |  | |  | |  | |  |  |  |
|  | KW-3-6 | 0 | Spring | 0.00125 | 84 | | 5.2×10^-11^ | | 0.00510 | | 0.25 | 0.1 | 3.9×10^-12^ |
|  | KW-3-12 | 0 | Spring | 0.00114 | 84 | | 5.2×10^-11^ | | 0.00510 | | 0.22 | 0.1 | 4.1×10^-12^ |

**
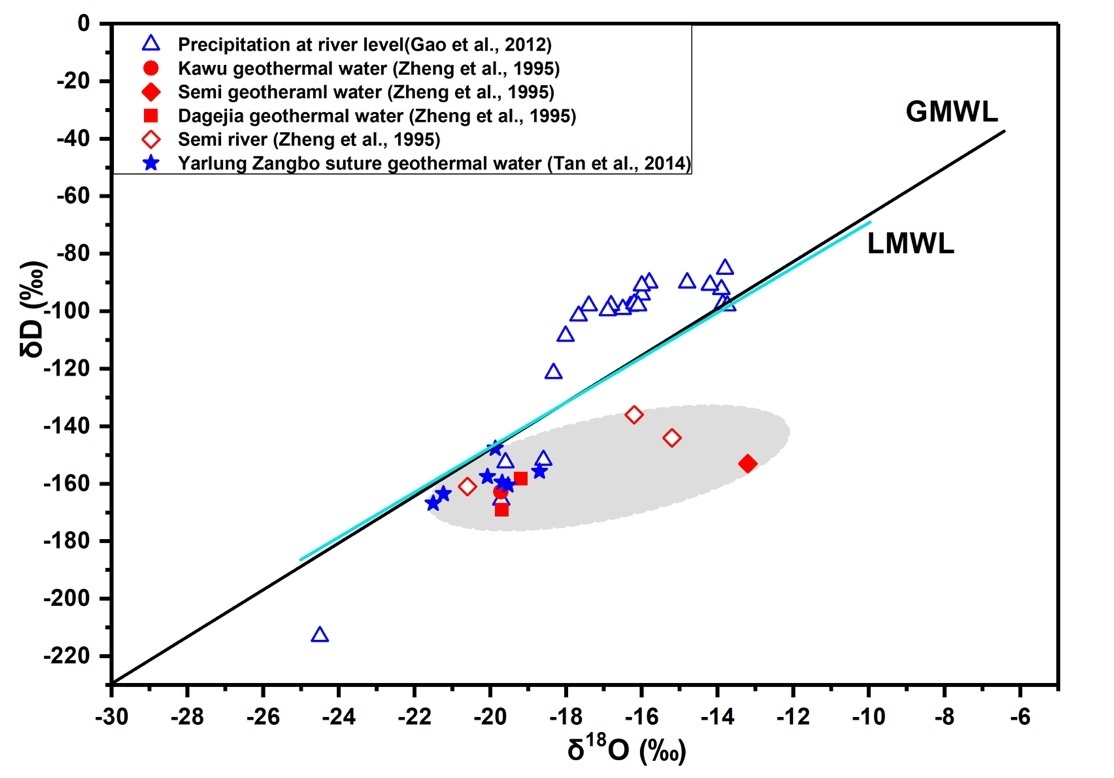
**

**Figure S1.** Correlation of δ^18^O vs. δD in different geothermal spring sites in the study area^1-3^. LMWL = local meteoric water line for the study area; GMWL = global meteoric water line with reference to Craig^4^.

**
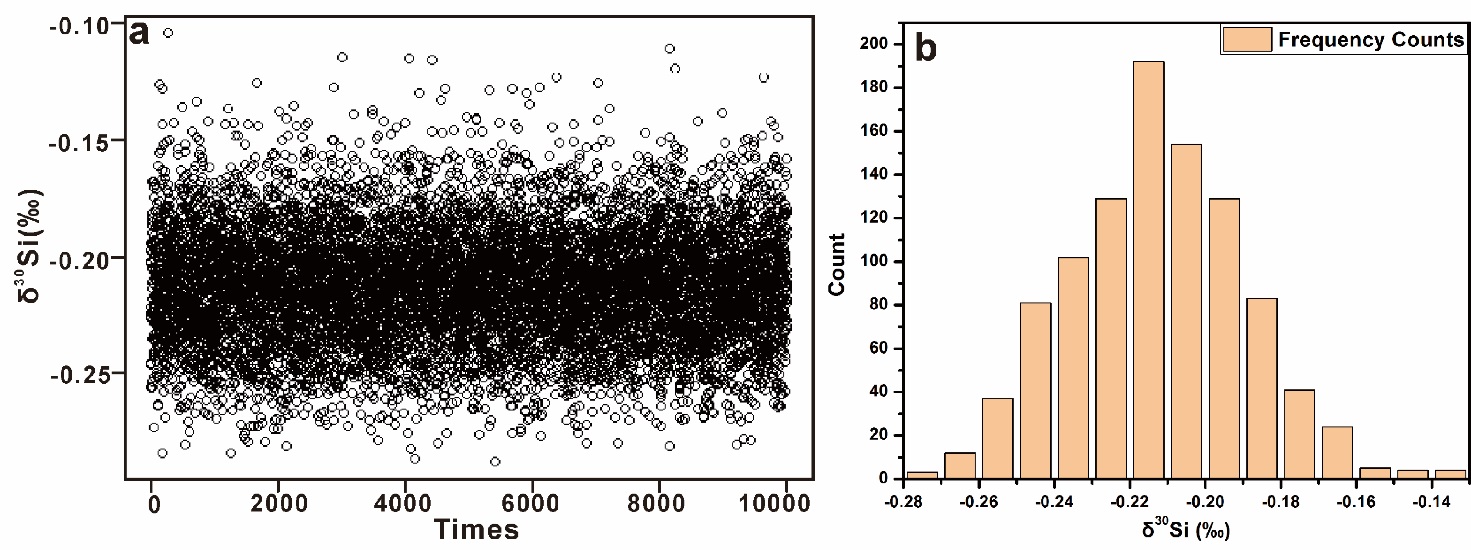
**

**Figure S2.** (a) Estimated δ^30^Si values of the host rocks of the geothermal reservoirs from an iterative calculation based on mass balance; (2) Frequency distribution of δ^30^Si values in the host rocks.

**
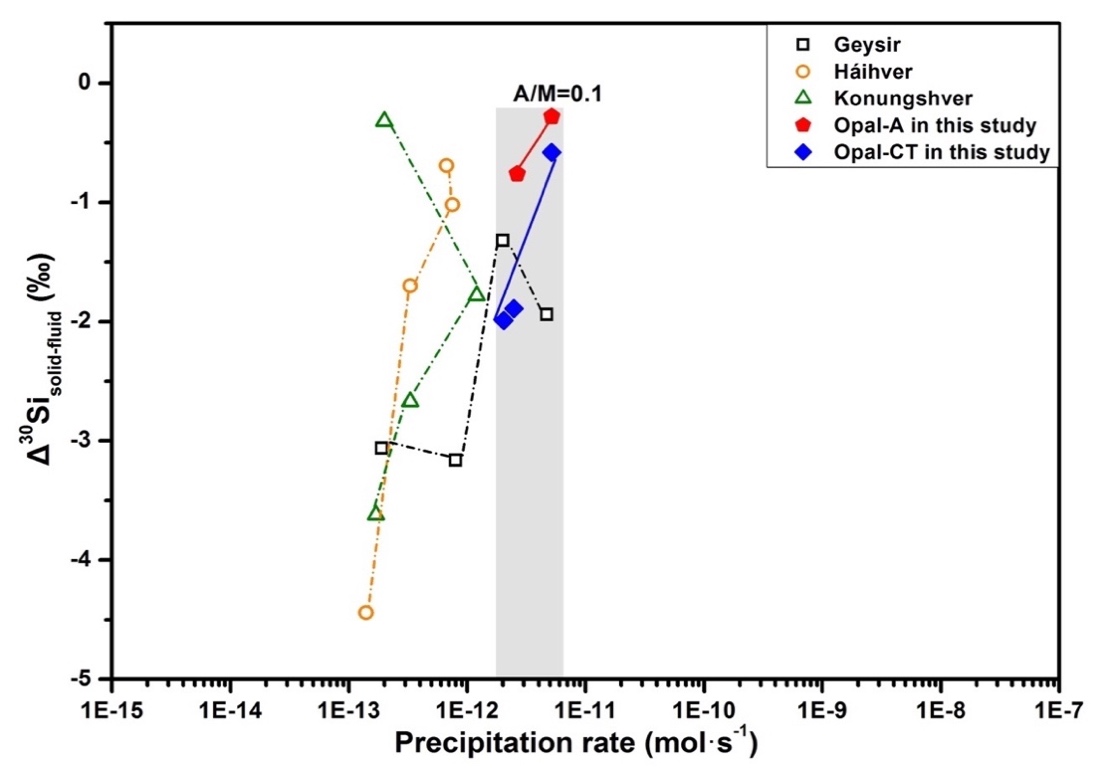
**

**Figure S3.** Dependence of silicon isotope fractionation on precipitation rate from this study and previous work^5^.

**Methods**

*Silicon isotope analysis*

The effects of other major anionic species (e.g. SO_4_^2-^, Cl^-^) on silicon isotope values were evaluated in this study. As observed in our experiment, the matrix effect from anionic species of Cl^-^ on the measured silicon isotopic compositions measured was not significant, which might be attributed to the relatively low ionization efficiency of Cl^-^ ions into Cl^+^ in plasma. This is consistent with a previous study by Ehlert et al. (2016)^6^, in which the concentration of Cl^-^ ranged up to 550 mM is one order of magnitude higher than our study (i.e. [Cl^-^] of 25 mM). The matrix effect of SO_4_^2-^ on the measured δ^30^Si values was checked by doping SO_4_^2-^ into the solution of the standard reference material GBW-04422. As shown in Figure S5, the measured δ^30^Si value measured remained in the recommended range of -2.79 ± 0.05 ‰ when the SO_4_^2-^/Si molar ratios were less than 3.13:1 mol/mol, but shifted negatively for higher SO_4_^2-^/Si ratios. The previous studies have reported that the offsets in δ^30^Si become significant for the SO_4_^2-^/Si molar ratios greater than 0.013 mol/mol, and reach up to ca. +1.4‰ at the SO_4_^2-^/Si molar ratios above ~0.13 mol/mol^7,8^. The discrepancy might be attributed to the differences in the sample introduction conditions (e.g., wet/dry plasma) and the shape of the spray chambers used in different laboratories. As the molar ratios of SO_4_^2-^/Si in geothermal water sampled in this study ranged from 0.19 to 2.67 mol/mol, the matrix effect from SO_4_^2-^ ions could be ignored.

**
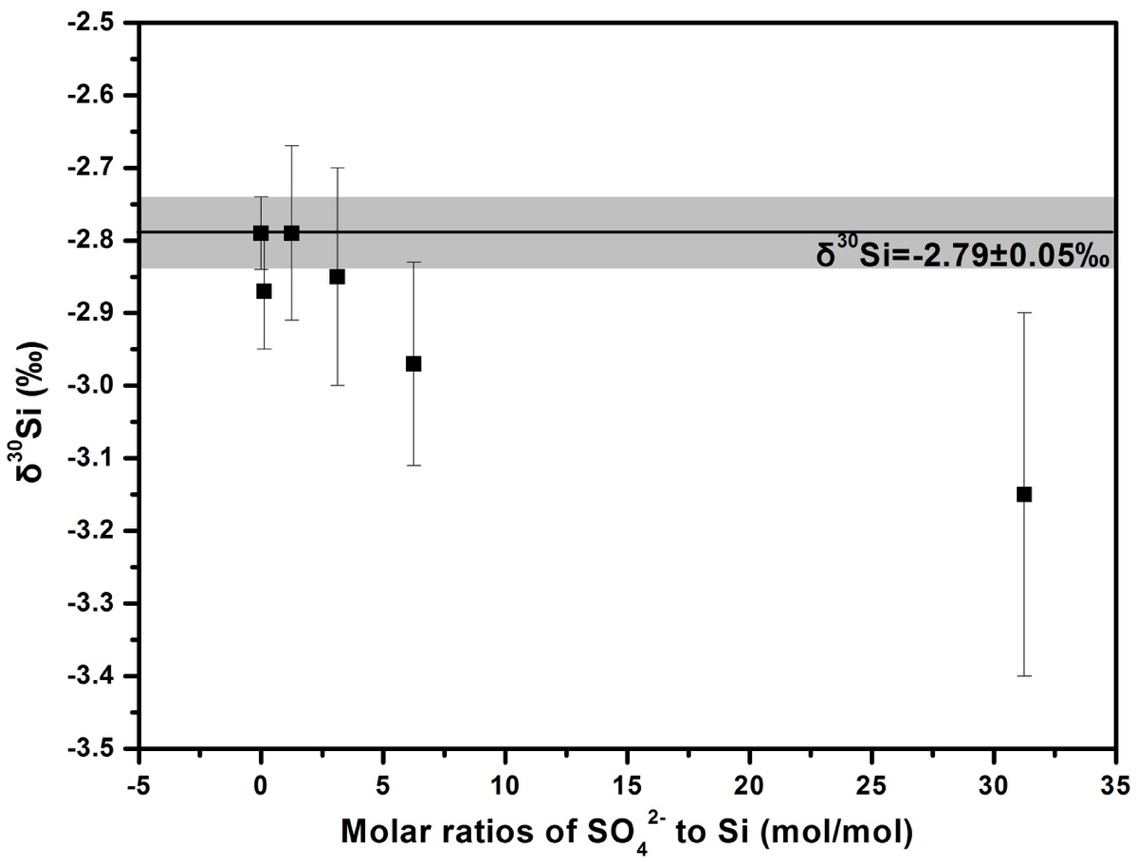
**

**Figure S4.** Influence of anionic species SO_4_^2-^ on measured δ^30^Si values.

**References**

1. Tan, H.B. et al. Understanding the circulation of geothermal waters in the Tibetan Plateau using oxygen and hydrogen stable isotopes. Appl. Geochem. 51, 23-32 (2014).

2. Zhao, H. et al. Deuterium excess record in a southern Tibetan ice core and its potential climatic implications. Clim. Dynam. 38, 1791-1803 (2012).

3. Gao, Z.Y., Wang, X.D. & Yin, G. Isotopic effect of runoff in the Yarlung Zangbo River. Chin. J. Geochem. 31, 309-314 (2012).

4. Craig, H. Isotopic variation in meteoric waters. Science 133, 1702-1703 (1961).

5. Geilert, S. et al. Silicon isotope fractionation during silica precipitation from hot-spring waters: evidence from the Geysir geothermal field, Iceland. Geochim. Cosmochim. Ac. 164, 403-427 (2015).

6. Ehlert, C. et al. Transformation of silicon in a sandy beach ecosystem: Insights from stable silicon isotopes from fresh and saline groundwaters. Chem. Geol. 440, 207-218 (2016).

7. van den Boorn, S.H. J. M.; Vroon, P.Z. & van Bergen, M.J. Sulfur-induced offsets in MC-ICP-MS silicon-isotope measurements. J. Anal. Atom. Spectrom. 24, 1111-1114 (2009).

8. Hughes, H.J. et al. Controlling the mass bias introduced by anionic and organic matrices in silicon isotopic measurements by MC-ICP-MS. J. Anal. Atom. Spectrom. 26, 1892-1896 (2011**)**
